# Alpha rhythm and Alzheimer’s disease: Has Hans Berger’s dream come true?

**DOI:** 10.1016/j.clinph.2025.02.256

**Published:** 2025-02-13

**Authors:** Claudio Babiloni, Xianghong Arakaki, Sandra Baez, Robert J. Barry, Alberto Benussi, Katarzyna Blinowska, Laura Bonanni, Barbara Borroni, Jorge Bosch Bayard, Giuseppe Bruno, Alessia Cacciotti, Filippo Carducci, John Carino, Matteo Carpi, Antonella Conte, Josephine Cruzat, Fabrizia D’Antonio, Stefania Della Penna, Claudio Del Percio, Pierfilippo De Sanctis, Javier Escudero, Giovanni Fabbrini, Francesca R. Farina, Francisco J. Fraga, Peter Fuhr, Ute Gschwandtner, Bahar Güntekin, Yi Guo, Mihaly Hajos, Mark Hallett, Harald Hampel, Lutfu Hanoğlu, Ira Haraldsen, Mahmoud Hassan, Christoffer Hatlestad-Hall, András Attila Horváth, Agustin Ibanez, Francesco Infarinato, Alberto Jaramillo-Jimenez, Jaeseung Jeong, Yang Jiang, Maciej Kamiński, Giacomo Koch, Sanjeev Kumar, Giorgio Leodori, Gang Li, Roberta Lizio, Susanna Lopez, Raffaele Ferri, Fernando Maestú, Camillo Marra, Laura Marzetti, William McGeown, Francesca Miraglia, Sebastian Moguilner, Davide V. Moretti, Faisal Mushtaq, Giuseppe Noce, Lorenzo Nucci, John Ochoa, Paolo Onorati, Alessandro Padovani, Chiara Pappalettera, Mario Alfredo Parra, Matteo Pardini, Roberto Pascual-Marqui, Walter Paulus, Vittorio Pizzella, Pavel Prado, Géraldine Rauchs, Petra Ritter, Marco Salvatore, Hernando Santamaria-García, Michael Schirner, Andrea Soricelli, John-Paul Taylor, Hatice Tankisi, Franca Tecchio, Stefan Teipel, Alpha Tom Kodamullil, Antonio Ivano Triggiani, Mitchell Valdes-Sosa, Pedro Valdes-Sosa, Fabrizio Vecchio, Keith Vossel, Dezhong Yao, Görsev Yener, Ulf Ziemann, Anita Kamondi

**Affiliations:** a Department of Physiology and Pharmacology “Vittorio Erspamer,” Sapienza University of Rome, Rome, Italy; b San Raffaele of Cassino, Cassino, (FR), Italy; c Cognition and Brain Integration Laboratory, Neurosciences, Huntington Medical Research Institutes, Pasadena, CA, USA; d Universidad de los Andes, Bogota, Colombia; e Global Brain Health Institute (GBHI), University of California, San Francisco, USA; f Trinity College Dublin, Dublin, Ireland; g Brain & Behaviour Research Institute and School of Psychology, University of Wollongong, Wollongong 2522, Australia; h Neurology Unit, Department of Medical, Surgical and Health Sciences, University of Trieste, Trieste, Italy; i Department of Biomedical Physics, Faculty of Physics, University of Warsaw, Poland; j Nalecz Institute of Biocybernetics and Biomedical Engineering, Warsaw, Poland; k Department of Medicine, Aging Sciences University G. d’Annunzio of Chieti-Pescara Chieti 66100 Chieti, Italy; l Neurology Unit, Department of Clinical and Experimental Sciences, University of Brescia, Brescia, Italy; m Molecular Markers Laboratory, IRCCS Istituto Centro San Giovanni di Dio Fatebenefratelli, Brescia 25125, Italy; n Facultad de Psicología, Universidad Autonoma de Madrid, Madrid, Spain; o Department of Human Neuroscience, Sapienza University of Rome, Rome, Italy; p Brain Connectivity Laboratory, Department of Neuroscience and Neurorehabilitation, IRCCS San Raffaele Roma, Rome, Italy; q Department of Theoretical and Applied Sciences, eCampus University, Novedrate, Como, Italy; r Clinical Neurophysiology, Royal Melbourne Hospital, Parkville, Melbourne, Australia; s IRCCS Neuromed, Pozzilli, Italy; t Latin American Brain Health Institute (BrainLat), Universidad Adolfo Ibañez, Santiago, Chile; u Department of Neuroscience, Imaging and Clinical Sciences, “G. d’Annunzio” University of Chieti and Pescara, Chieti, Italy; v Institute for Advanced Biomedical Technologies (ITAB), “G. d’Annunzio” University of Chieti and Pescara, Chieti, Italy; w Albert Einstein College of Medicine, Department of Neurology, Bronx, NY 10461, USA; x Institute for Imaging, Data and Communications, School of Engineering, University of Edinburgh, UK; y The University of Chicago Division of the Biological Sciences 5841 S Maryland Avenue Chicago, IL 60637, USA; z Global Brain Health Institute (GBHI), Trinity College Dublin, Ireland; aa Engineering, Modeling and Applied Social Sciences Center, Federal University of ABC, Santo André, Brazil; ab Department of Neurology, Hospitals of the University of Basel, Basel, Switzerland; ac Department of Biophysics, School of Medicine, Istanbul Medipol University, Istanbul, Turkey; ad Research Institute for Health Sciences and Technologies (SABITA), Istanbul Medipol University, Istanbul, Turkey; ae Department of Neurology, Shenzhen People’s Hospital and The First Affiliated Hospital, Southern University of Science and Technology, Shenzhen, China; af Shenzhen Bay Laboratory, Shenzhen, China; ag Tianjin Huanhu Hospital, Tianjin, China; ah Cognito Therapeutics, Cambridge, MA, USA; ai Department of Comparative Medicine, Yale University School of Medicine, New Haven, CT, USA; aj Human Motor Control Section, Medical Neurology Branch, National Institute of Neurological Disorders and Stroke, National Institutes of Health, Building 10, Room 7D37, 10 Center Drive, Bethesda, MD 20892-1428, USA; ak Sorbonne University, Alzheimer Precision Medicine, AP-HP, Pitié-Salpêtrière Hospital, Boulevard de l’hôpital, F-75013 Paris, France; al Department of Neurology, School of Medicine, Istanbul Medipol University, Istanbul, Turkey; am Department of Neurology, Oslo University Hospital, Oslo, Norway; an MINDIG, F-35000 Rennes, France; ao School of Science and Engineering, Reykjavik University, Reykjavik, Iceland; ap Neurocognitive Research Centre, Nyírő Gyula National Institute of Psychiatry and Addictology, Budapest, Hungary; aq Department of Anatomy, Histology and Embryology, Semmelweis University, Budapest, Hungary; ar Research Centre for Natural Sciences, HUN-REN, Budapest, Hungary; as Cognitive Neuroscience Center, Universidad de San Andrés, Victoria, Buenos Aires, Argentina; at Rehabilitation Bioengineering Laboratory, IRCCS San Raffaele Roma, Rome, Italy; au Centre for Age-Related Medicine (SESAM), Stavanger University Hospital, Stavanger, Norway; av Grupo de Neurociencias de Antioquia (GNA), Universidad de Antioquia, Medellín, Colombia; aw Department of Brain and Cognitive Sciences, Korea Advanced Institute of Science & Technology (KAIST), Daejeon 34141, South Korea; ax Aging Brain and Cognition Laboratory, Department of Behavioral Science, College of Medicine, University of Kentucky, Lexington, KY, USA; ay Sanders Brown Center on Aging, College of Medicine, University of Kentucky, Lexington, KY, USA; az Human Physiology Unit, Department of Neuroscience and Rehabilitation, University of Ferrara, Ferrara, Italy; ba Experimental Neuropsychophysiology Laboratory, IRCCS Santa Lucia Foundation, Rome, Italy; bb Department of Psychiatry, Faculty of Medicine, University of Toronto, Toronto, ON, Canada; bc Real World Evidence & Medical Value, Global Medical Affairs, Neurology, Eisai Inc., New Jersey, USA; bd Oasi Research Institute - IRCCS, Troina, Italy; be Center For Cognitive and Computational Neuroscience, Complutense University of Madrid, Spain; bf Department of Psychology, Catholic University of Sacred Heart, Milan, Italy; bg Memory Clinic, Foundation Policlinico Agostino Gemelli IRCCS, Rome, Italy; bh Department of Engineering and Geology, “G. d’Annunzio” University of Chieti and Pescara, Pescara, Italy; bi Department of Psychological Sciences & Health, University of Strathclyde, Graham Hills Building, 40 George Street, Glasgow, UK; bj Department of Neurology, Massachusetts General Hospital and Harvard Medical School, Boston, MA, USA; bk Alzheimer’s Rehabilitation Operative Unit, IRCCS Istituto Centro San Giovanni di Dio Fatebenefratelli, 25125 Brescia, Italy; bl School of Psychology, University of Leeds, Leeds, UK; bm NIHR Leeds Biomedical Research Centre, Leeds, UK; bn IRCCS Synlab SDN, Naples, Italy; bo Neurophysiology Laboratory GNA-GRUNECO. Universidad de Antioquia, Antioquia, Colombia; bp Department of Continuity of Care and Frailty, Neurology Unit, ASST Spedali Civili Hospital, Brescia, Italy; bq Neurobiorepository and Laboratory of Advanced Biological Markers, University of Brescia, ASST Spedali Civili Hospital, Brescia, Italy; br Laboratory of Digital Neurology and Biosensors, University of Brescia, Brescia, Italy; bs Brain Health Center, University of Brescia, Brescia, Italy; bt Department of Neurosciences, Rehabilitation, Ophthalmology, Genetics, Maternal and Child Health, (DINOGMI), University of Genoa, Genoa, Italy; bu IRCCS Ospedale Policlinico San Martino, Genova, Italy; bv The KEY Institute for Brain-Mind Research, University Hospital of Psychiatry, Zurich, Switzerland; bw Department of Neurology, Ludwig-Maximilians University Munich, Munich, Germany; bx University Medical Center Göttingen, Göttingen, Germany; by Escuela de Fonoaudiología, Facultad de Odontología y Ciencias de la Rehabilitación, Universidad San Sebastián, Santiago, Chile; bz Normandie Univ, UNICAEN, INSERM, U1237, PhIND “Physiopathology and Imaging of Neurological Disorders”, NeuroPresage Team, GIP Cyceron, 14000 Caen, France; ca Berlin Institute of Health, Charité, Universitätsmedizin Berlin, Berlin, Germany; cb Department of Neurology with Experimental Neurology, Charité, Universitätsmedizin Berlin, Berlin, Germany; cc Bernstein Focus State Dependencies of Learning and Bernstein Center for Computational Neuroscience, Berlin, Germany; cd Einstein Center for Neuroscience Berlin, Berlin, Germany; ce Einstein Center Digital Future, Berlin, Germany; cf Pontificia Universidad Javeriana (PhD Program in Neuroscience), Bogotá, Colombia; cg Center of Memory and Cognition Intellectus, Hospital Universitario San Ignacio Bogotá, San Ignacio, Colombia; ch Department of Medical, Movement and Wellbeing Sciences, University of Naples Parthenope, Naples, Italy; ci Translational and Clinical Research Institute, Newcastle University, Newcastle upon Tyne, UK; cj Department of Clinical Neurophysiology, Aarhus University Hospital, Aarhus, Denmark; ck Consiglio Nazionale delle Ricerche (CNR), Istituto di Scienze e Tecnologie della Cognizione (ISTC), Roma, Italy; cl German Center for Neurodegenerative Diseases (DZNE) Rostock, Rostock, Germany; cm Department of Bioinformatics, Fraunhofer Institute for Algorithms and Scientific Computing (SCAI), Sankt Augustin, Germany; cn Neurophysiology of Epilepsy Unit, National Institute of Neurological Disorders and Stroke, National Institutes of Health, Bethesda, MD 20892, USA; co Cuban Center for Neuroscience, Havana, Cuba; cp The Clinical Hospital of Chengdu Brain Science Institute, University of Electronic Science and Technology of China, Chengdu, China; cq Department of Neurology, David Geffen School of Medicine at University of California, Los Angeles, CA, USA; cr Department of Neurology, Faculty of Medicine, Dokuz Eylül University, İzmir, Turkey; cs Izmir Biomedicine and Genome Center, Izmir, Turkey; ct Department of Neurology & Stroke, University of Tübingen, Tübingen, Germany; cu Hertie Institute for Clinical Brain Research, University of Tübingen, Tübingen, Germany; cv Department of Neurosurgery and Neurointervention and Department of Neurology, Semmelweis University, Budapest, Hungary

**Keywords:** Hans Berger, Resting-State Electroencephalographic (rsEEG), Rhythms, Delta, Theta, and Alpha Rhythms, Alzheimer’s Disease (AD), Mild Cognitive Impairment (MCI), Biomarkers

## Abstract

In this “centenary” paper, an expert panel revisited Hans Berger’s groundbreaking discovery of human restingstate electroencephalographic (rsEEG) alpha rhythms (8–12 Hz) in 1924, his foresight of substantial clinical applications in patients with “senile dementia,” and new developments in the field, focusing on Alzheimer’s disease (AD), the most prevalent cause of dementia in pathological aging.

Clinical guidelines issued in 2024 by the US National Institute on Aging-Alzheimer’s Association (NIA-AA) and the European Neuroscience Societies did not endorse routine use of rsEEG biomarkers in the clinical workup of older adults with cognitive impairment. Nevertheless, the expert panel highlighted decades of research from independent workgroups and different techniques showing consistent evidence that abnormalities in rsEEG delta, theta, and alpha rhythms (< 30 Hz) observed in AD patients correlate with wellestablished AD biomarkers of neuropathology, neurodegeneration, and cognitive decline. We posit that these abnormalities may reflect alterations in oscillatory synchronization within subcortical and cortical circuits, inducing cortical inhibitory-excitatory imbalance (in some cases leading to epileptiform activity) and vigilance dysfunctions (e.g., mental fatigue and drowsiness), which may impact AD patients’ quality of life.

Berger’s vision of using EEG to understand and manage dementia in pathological aging is still actual.

## 1. Background and aim of this article

**Fig. 1.**
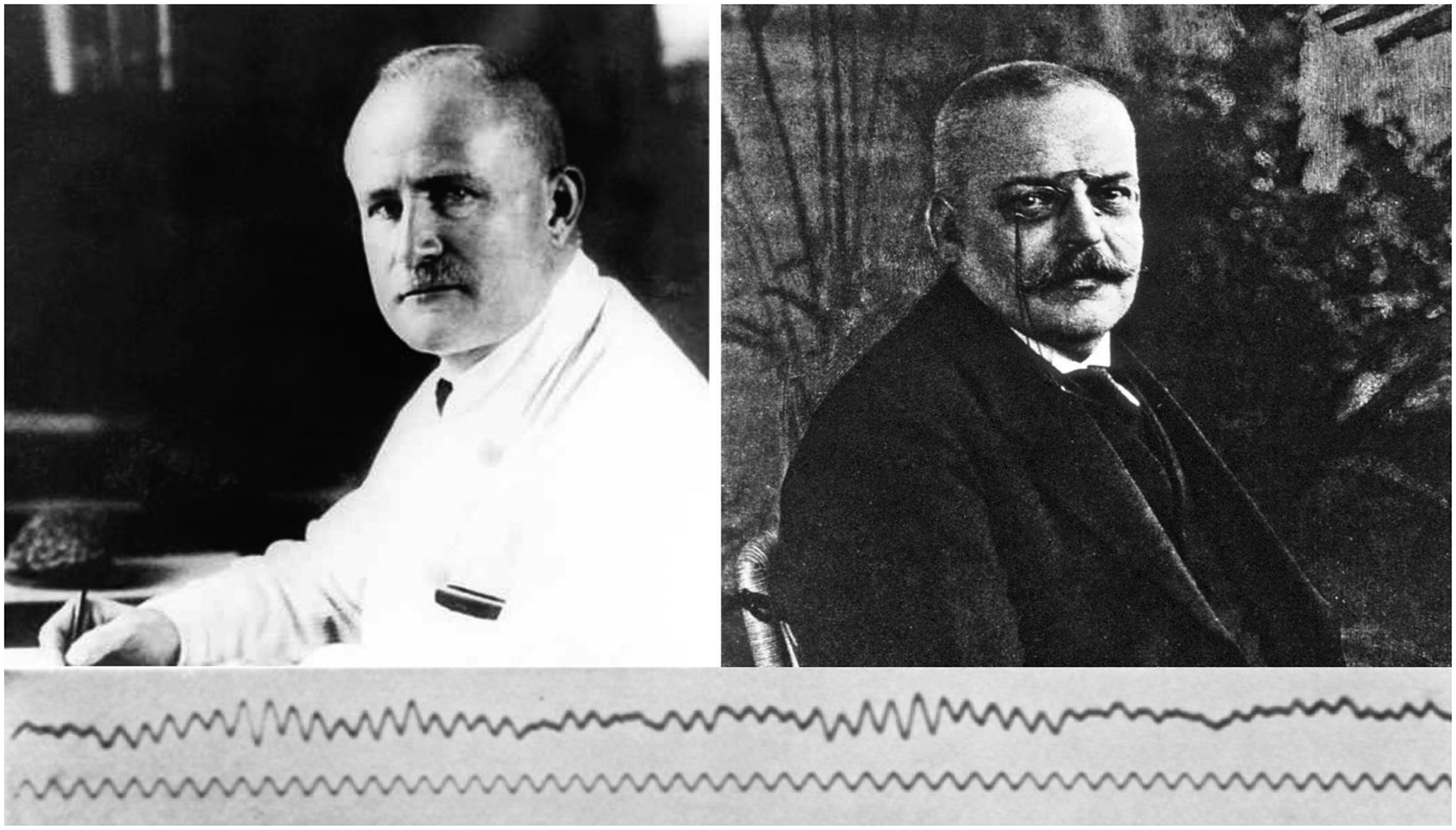
Upper left: The German Psychiatrist Hans Berger (1873–1941) as portrayed in 1930. At that time, Hans Berger had already recorded tens of resting-state electroencephalographic (rsEEG) recordings from the scalp, skull, dura mater, and cerebral cortex in humans in conditions of eyes closed and open, sensory stimulations, and cognitive tasks. The first article on human EEG was published in 1929 (Berger, 1929). This Berger’s photo is available as uncredited, public domain, thanks to Wikimedia Commons: File “HansBerger Univ Jena.jpeg.”- https://commons.wikimedia.org/w/index.php?curid=12160449. Upper right: The German Psychiatrist and Neuropathologist Alois Alzheimer (1864–1915) as portrayed. Alzheimer’s was a colleague of famous German Psychiatrist Emil Kraepelin. Alzheimer published the first case of “presenile dementia,” after named Alzheimer’s disease by Kraepelin. This Alzheimer’s photo is available as uncredited, public domain, thanks to Wikimedia Commons: File “Alois Alzheimer 002.jpg.” https://commons.wikimedia.org/wiki/File:Alois_Alzheimer_002.jpg. Bottom: An EEG (top) activity recorded from the scalp of Berger’s son, Klaus (15 years old). It is the first scalp-recorded EEG activity published by Berger in a scientific paper (Berger, 1929), so it is the first EEG trace ever published! This EEG activity was recorded while Klaus was in a condition of resting-state eyes closed. Berger put a 10-Hz sinusoid under that rsEEG trace to emphasize that the dominant EEG oscillatory activity had a frequency of around 10 Hz. In Berger’s paper of 1929, he named that dominant EEG activity as “primary.” One year later, he named it “alpha waves” (Berger, 1930). This Berger’s picture is available as uncredited, public domain, thanks to Wikimedia Commons: File “1st-eeg.png”- https://commons.wikimedia.org/wiki/File:1st-eeg.png.

Hans Berger (1873–1941; Fig. 1), a Professor of Psychiatry at the University of Jena, Germany, and Director of its psychiatry clinic, pursued a visionary goal in the early 1900 s: to uncover the relationship between mental disorders and abnormal brain activity, detectable through heat and electrical currents. This ambition led to his groundbreaking discovery in 1924, when he recorded and described human brain electrical activity, coining the term “electroencephalogram (EEG).”

A century later, an expert panel revisited a key aspect of Berger’s “scientific dream”: the potential role of EEG in understanding the neurophysiological underpinnings of what was previously termed “senile dementia” (Berger, 1938). The expert panel presents the collective perspectives of neurologists, neuroscientists, psychiatrists, clinical neurophysiologists, psychologists, computer scientists, and biophysicists from several prominent organizations, including the Special Interest Group on “Advanced EEG/MEG Techniques in Clinical Neurophysiology” of the International Federation of Clinical Neurophysiology (https://www.ifcn.info/get-involved/special-interest-groups/advanced-eeg-meg-techniques-in-clinicalneurophysiology), the PDWAVES Consortium (https://www.pdwaves.eu/), the Electrophysiology Professional Interest Area of the Alzheimer’s Association International Society to Advance Alzheimer’s Research and Treatment (https://istaart.alz.org/PIAs), Latin America and the Caribbean Consortium on Dementia (LAC-CD; https://lac-cd.org/), EuroLad EEG Consortium (https://bit.ly/4ctCJzi), the ReDLat Consortium (https://red-lat.com/), eBRAIN-Health (https://ebrain-health.eu/home.html), EBRAINS (https://www.ebrains.eu/), AI-mind (https://www.ai-mind.eu/), and the Global Brain Consortium (https://globalbrainconsortium.org/). The views and considerations expressed herein are of the co-authors of this “centenary” paper and do not necessarily reflect the official positions of the above Consortia as a whole.

To explore this, the expert panel considered Alzheimer’s disease (AD) as a showcase. It is the most prevalent cause of progressive cognitive decline and disabilities in the activity of daily living in older people affected by pathological brain aging, affecting many millions of older individuals worldwide (Lanctôt et al., 2024). AD is a progressive brain proteinopathy, triggered and aggravated by regional brain amyloidosis and tauopathy, leading to primary neurodegenerative pathology with cognitive deficits ranging from mild cognitive impairment (ADMCI) to dementia (ADD) (Lanctôt et al., 2024).

Specifically, the expert panel addressed the following questions: has Hans Berger’s vision for EEG’s role in advancing our understanding of AD (as a model of dementia in pathological brain aging) come true? A century later, what role does the analysis of resting-state EEG (rsEEG) rhythms play in the clinical research and evaluation of AD patients with mild cognitive impairment (ADMCI) and mild-to-moderate dementia (ADD)? The expert panel answers these questions based on decades of research, positing that changes in rsEEG rhythms at Berger’s frequencies (< 30 Hz) in ADMCI and ADD patients may partially reflect alterations in cortical inhibitory/excitability balance (in some cases leading to epileptiform activity) and vigilance regulation. These alterations would be associated with non-cognitive symptoms such as mental fatigue, difficulties in maintaining concentration (watching TV and reading), daytime drowsiness, and sleepiness with morning naps, which significantly impact the quality of life of AD patients. Notably, the related rsEEG measures may be a useful reference for the treatment of those disease correlates and manifestations.

From a methodological perspective, this paper offers a curated, reflective examination of EEG rhythms in ADMCI and ADD patients, grounded on an arbitrary selection of experimental studies and reviews. It is not intended as a systematic review. Furthermore, the expert panel did not address the identification of the optimal rsEEG monitoring, predictive, and therapy response biomarkers for clinical application in ADMCI and ADD patients, as this will require an international effort that implements a well-designed comparative experimental study using the most effective EEG techniques. Moreover, this paper is not a systematic excursus of Berger’s discoveries, grand vision, and history. For this purpose, we encourage readers to read previous, excellent commemorative papers (Caeira et al., 2023; Gloor and Berger lecture, 1994, 1969; Stone and Hughes, 2013), including a recent initiative surveying more than 500 experts to evaluate the significant influence of EEG discovery on our understanding of the brain and behavior and perspectives over the next century (Mushtaq et al., 2024).

Notably, the present paper is focused on spontaneous EEG (rsEEG) rhythms in ADMCI and ADD patients. Along this line, it does not cover other promising areas of EEG research. For example, the paper does not consider the analysis of rsEEG “microstates,” which are characterized by dynamic changes over time in certain spatial patterns of scalp EEG voltage during resting-state conditions. EEG microstates have been shown to be abnormal in AD patients (e.g., (Smailovic and Jelic, 2019)) and modulated by non-invasive brain stimulation (e.g., (Hanoglu et al., 2022)). Furthermore, the paper does not consider stimulus-evoked and event-related potentials or oscillations despite emerging, interesting results (Babiloni et al., 2020b; Güntekin et al., 2022). Moreover, the EEG responses evoked by non-invasive brain stimulations (e.g., transcranial magnetic stimulation) unveiled posterior cortical overexcitability in ADD patients (Casula et al., 2023; Maiella et al., 2024) as a promising target for non-invasive magnetic stimulation used as intervention (Koch et al., 2022). Finally, we recommend reading papers on neurophysiological oscillatory mechanisms regulating cortical excitability as revealed by multimodal transcranial magnetic stimulation and simultaneous recording of rsEEG rhythms (Belardinelli et al., 2021; Zrenner et al., 2023).

## 2. The discovery of human EEG

Hans Berger conducted the first human EEG recording on July 6, 1924, at the Psychiatry Clinic in Jena, Germany. This historic event took place during a neurosurgical operation on a 17-year-old boy, referred to as Patient K, performed by neurosurgeon Nikolai Guleke. Berger placed two electrodes into a breach in the skull, overcoming the high electrical resistance of the scalp and skull. The EEG signals were captured using an Edelmann string galvanometer. Since electronic data storage was not available at the time, the EEG activity was displayed on an oscilloscope and recorded on photographic paper. However, due to the low sensitivity of the galvanometer, these early measurements provided only preliminary results.

In 1926, Berger began using a more sensitive Siemens double-coil galvanometer, which enabled him to achieve more consistent and accurate EEG recordings. By 1929, he published his first article documenting ongoing human EEG activity during wakefulness, using recordings from the scalp, skull, and dura mater (Berger, 1929). This publication was based on EEG data from around 40 individuals, both healthy and those with skull defects.

Over the following years (1929–1938), Berger performed numerous EEG recordings from different brain regions in healthy volunteers, individuals with skull defects, and patients with psychiatric disorders. His experiments investigated EEG activity during various conditions, including quiet wakefulness, sleep, narcosis, and cognitive tasks, revealing corresponding changes in EEG patterns in healthy subjects (Berger, 1933, 1931, 1930, 1929). In patients with neurological and psychiatric symptoms, most experiments were conducted in a resting state, eyes-closed condition, though some also assessed the effects of psychoactive drugs on EEG activity (Berger, 1938, 1933, 1931, 1930, 1929).

Berger was the first to identify spontaneous EEG rhythms associated with a condition of psychophysiological relaxation and mind wandering without any substantial goal-directed flow of thoughts. He called it a “passive condition” to emphasize the lack of any conscious control of the direction of thoughts (Berger, 1938). Today, this psychophysiological mode is called a “resting-state” condition. In this sense, he was the first to introduce the investigation of the resting-state condition in brain research and clinical applications. Nowadays, this condition represents the most studied paradigm in clinical neurosciences using EEG, its magnetic counterpart (i.e., magnetoencephalography, MEG), positron emission tomography of brain glucose metabolism (FDGPET), functional magnetic resonance imaging (fMRI), and others. It should be remarked that the resting-state condition is not an “artificial” psychophysiological condition in settings of brain research. It occurs in everyday life to avoid the transition from quiet vigilance to drowsiness and sleep. The resting-state condition can also be considered as a sort of “baseline mode” between “executive modes” of the brain during the goaloriented elaboration of external or internal sensory stimuli and decision-making processes induced by internal plans or emerging environmental events. Notably, the evaluation of the resting-state brain mode may also predict brain processes regulating quiet vigilance in ecological conditions, such as passively watching TV programs without stressful content (e.g., documentaries, etc.) or listening to relaxing music, which are conditions that are relatively similar to the experimental resting-state condition and may be characterized by several intermingled phases of mind wandering and active information processing. We refer to the resting-state mode in this ecological perspective.

In 1930, Berger identified the prominent 8–12 Hz rsEEG rhythms as “alpha waves,” which appeared when participants had their eyes closed and were in a relaxed psychophysiological state. He observed that these “alpha waves” disappeared or desynchronized and were replaced by lower-amplitude, higher-frequency “beta waves” (14–30 Hz) when participants opened their eyes, received sensory stimulation, or engaged in cognitive tasks, such as counting (Berger, 1930).

Berger demonstrated that spontaneous EEG “alpha and beta waves” did not significantly correlate with physiological functions, such as heart rate, concurrent electrocardiographic activity, respiratory cycles, or muscle tension, ruling out the possibility that they were artifacts of other bodily functions. He proposed that these waves reflected neurophysiological control mechanisms regulating overall brain activity in relation to cortical arousal and mental states, including fluctuations in vigilance, attention, and the flow of thought. Specifically, he suggested that “alpha waves” were associated with automated control of spontaneous thought flow, while “beta waves” were linked to active mental processes (Berger, 1938).

Berger further theorized that deviations from typical rsEEG patterns could signal underlying brain pathological processes, providing a novel method for diagnosing and monitoring mental disorders through neurophysiological biomarkers. His observations included irregular rsEEG “alpha waves” slowing in frequency to about 5 Hz in individuals with cognitive deficits, including those in the stages of what was then termed senile dementia (Berger, 1938). These insights laid the foundation for modern approaches to developing neurophysiological biomarkers for aging-related neurodegenerative diseases, such as AD, where early detection and monitoring over time of the disease through biomarkers grounds precision medicine.

Berger’s discovery of rsEEG rhythms initially faced significant skepticism from the scientific community. His findings were met with doubt, as the validity of human EEG was questioned. During the early 1930 s, many British and American researchers lacked access to Berger’s German publications on human EEG. As a result, Berger’s methods and interpretations were heavily criticized. This skepticism was partly due to the novelty of the concept and the technological limitations of the time, which made it difficult for others to replicate his results consistently. Additionally, many scientists doubted that meaningful electrical activity could be recorded from the scalp and brain’s surface, given the huge complexity of electrochemical transmissions across neural fibers (Gloor, 1969).

A turning point came in 1934 when British neurophysiologist Edgar Douglas Adrian, a Nobel laureate for his work on nerve function, and engineer Brian Matthews successfully confirmed Berger’s findings (Adrian and Matthews, 1934). Using a similar setup to Berger’s experiments, with a frontal-posterior electrode pair and a single recording channel, they replicated his EEG results. This empirical validation established Berger’s work as accurate and profoundly significant for understanding human brain neurophysiology. Adrian and Matthews demonstrated that eyes-closed rsEEG “alpha waves” were a consistent and reproducible phenomenon, cementing EEG’s credibility in neuroscience.

In 1935, Herbert Jasper expanded on Berger’s findings and published the first rsEEG rhythms recorded in North America (Jasper and Carmichael, 1935). Jasper’s work was pivotal in establishing the reliability of EEG activity and demonstrating its potential for advancing the understanding of brain function across various states of vigilance and sensory stimulation. By refining EEG methods, including the use of multiple rsEEG channels, Jasper linked specific EEG rhythms to distinct cognitive and neurological functions, bridging the gap between basic neuroscience and clinical practice. Notably, Jasper was the first to document that EEG alpha rhythms remained blocked for nearly a second after a visual flash stimulus was turned off, indicating the influence of both the stimulus and the related iconic memory.

In the late 1930s, American neurologist Frederick Lemere conducted extensive rsEEG recordings and published findings on hundreds of healthy volunteers and patients with conditions such as depression, schizophrenia, epilepsy, and what was called “senile dementia.” Lemere’s work confirmed Berger’s observations of frequency slowing in rsEEG activity in these patients, compared to Berger’s “alpha waves,” and even reported pathological replacement of “alpha waves” with oscillatory activity below 4 Hz (Lemere, 1939, 1936). These studies reinforced the significance of EEG as a tool for detecting, diagnosing, and understanding neurological and psychiatric disorders, further validating Berger’s pioneering contributions.

## 3. Berger’s rsEEG alpha rhythms a century after their discovery

Berger’s rsEEG “alpha and beta waves” are now commonly referred to as alpha and beta “rhythms” or “activity” to emphasize that they are not transient phenomena and may be observed in rsEEG recordings over minutes. Notably, the original meaning of the rsEEG activity in relation to the participant’s psychophysiological condition is still valid. However, research has expanded our knowledge about the underlying neurophysiological mechanisms and their relationship with brain processes and status. This knowledge, summarized in the following, is the basis for the core thesis of this paper.

Posterior rsEEG alpha rhythms in healthy adults are widely recognized as reflecting the regulation of neuromodulatory subcortical ascending systems involved in cortical arousal and vigilance during quiet wakefulness (Pfurtscheller and Lopes Da Silva, 1999; Wan et al., 2019). Concerning the relationship between rsEEG alpha rhythms and vigilance function, previous studies have shown a reduction in the amplitude of posterior rsEEG alpha and an increase in the amplitude of rsEEG delta (< 4 Hz) rhythms following one night of sleep deprivation (Del Percio et al., 2019). Furthermore, a transition from rsEEG alpha to theta (4–7 Hz) and delta rhythms has been related to omitted responses during a boring continuous reaction time task performed with eyes closed (Jagannathan et al., 2018). Along the same line, a slowing in the frequency of rsEEG alpha rhythms across aging has been related to diminished sustained attention for about 10 min (Campbell et al., 2024). Another research line revealed a relationship between rsEEG alpha rhythms and vigilance function by comparing the rsEEG rhythms recorded before and after a visual motion direction discrimination task performed for 18 min (Kavcic et al., 2021). Cognitively unimpaired older adults showed a substantial amplitude reduction of posterior rsEEG rhythms after the task, possibly related to mental fatigue and reduced vigilance (Kavcic et al., 2021).

Other studies support the relationship between rsEEG alpha rhythms and cortical arousal. A negative correlation between the amplitude of rsEEG alpha rhythms and skin conductance levels, a marker of autonomic arousal, has been demonstrated (Barry et al., 2020). Furthermore, brief transcranial vagal nerve stimulation in healthy adults, compared to sham stimulations, causes transient dilation as a sign of increased autonomic arousal and attenuation of occipital rsEEG alpha rhythms as a reflection of increased cortical excitation (Sharon et al., 2021). This aligns with the known effects of such stimulation on the nucleus tractus solitarius in the brainstem and, subsequently, the locus coeruleus, a key part of the subcortical arousal system (Joshi et al., 2016; Sharon et al., 2021). Additionally, transcranial static magnetic field stimulation applied for inhibiting the occipital cortex has been shown to result in a localized increase in rsEEG alpha rhythms, as a neurophysiological mechanism underpinning cortical inhibition, and a concurrent reduction in visual search performance during a separate session (Gonzalez-Rosa et al., 2015). Moreover, transcranial magnetic stimulation of the dorsal premotor cortex produces weaker blood oxygen level-dependent (BOLD) activity of the bilateral cortico-subcortical motor systems (striatum-thalamus), as observed in resting-state fMRI (rsfMRI), when released during periods of strong rsEEG alpha rhythms (Peters et al., 2020). Finally, a positive association between rsEEG alpha rhythms and rs-fMRI BOLD activity in the thalamus, along with a predominantly negative association with BOLD activity in posterior cerebral areas related to visual and attentional processes, has also been observed during quiet wakefulness (De Munck et al., 2007; Knaut et al., 2019; Laufs et al., 2006; Olbrich et al., 2009). A century after Berger’s pioneering work, neurophysiological research on EEG rhythms summarized above supports the concept that rsEEG alpha rhythms negatively reflect the regulation of cortical arousal and vigilance and may be used to probe those aspects of brain (dys)functions in ADMCI and ADD patients.

## 4. Clinical guidelines of Alzheimer’s disease (AD) biomarkers a century after Berger’s discovery

What about the use of rsEEG rhythms as biomarkers in the assessment of AD patients? A century after Berger discovered human EEG, two prestigious international initiatives published criteria for using biomarkers in the AD continuum, ranging from ADMCI to ADD status. The US National Institute on Aging and Alzheimer’s Association (NIA-AA) updated (Jack et al., 2024) its theoretical framework for the neurobiological diagnosis of AD, originally outlined in the “Research Framework: Toward a Biological Definition of Alzheimer’s Disease” (Jack et al., 2018) and, subsequently, proposed to be extended (Hampel et al., 2021). The update reaffirms that AD diagnosis should be based on biomarkers of amyloidosis (A) and tauopathy (T), detectable through in-vivo measurements via cerebrospinal fluid (CSF), blood plasma, or neuroimaging (tau positron emission tomography, tau PET). Furthermore, the framework suggests that AD can be diagnosed based on these biomarkers, regardless of clinical symptoms, across the continuum from asymptomatic individuals to those with subjective cognitive decline (SCD), ADMCI, or ADD (Jack et al., 2024, 2018). The course of the clinical disease manifestations is carefully evaluated by standard neuropsychological tests of the core cognitive domains, such as episodic and short-term verbal and spatial memory, frontal executive functions (e.g., working memory, attention, refraining of impulsive responses), verbal fluidity, spatial skills, etc. Furthermore, the autonomy and independence of instrumental and non-instrumental activities of daily living are carefully evaluated for the diagnosis of dementia. In the revised framework, biomarkers of neurodegeneration (e.g., fluid biomarkers, structural MRI, and glucose metabolism PET) continue to play a central role. In the revised framework, there is an expanded focus on non-AD-specific biomarkers (e.g., neuroinflammation, cerebrovascular lesions, neuropathological comorbidities, etc.) that may enhance prognostication of the disease course and the assessment of therapeutic response (Jack et al., 2024). EEG techniques were just mentioned as a potential tool to unveil synaptic dysfunctions and functional connectivity but were not included in the actual biomarker panel for assessing ADMCI and ADD patients.

Similarly, a European multidisciplinary task force of 22 experts from 11 scientific societies developed a patient-centered diagnostic workflow for biomarker testing in individuals with MCI or mild-to-moderate dementia to determine the underlying neurobiological, etiological diagnosis (Frisoni et al., 2024). Using a Delphi consensus procedure, they identified 11 clinical syndromes, including AD, based on clinical history, examination, neuropsychology, blood and CSF tests, and neuroimaging. EEG biomarkers were recommended at the first step of the clinical workup only in cases where MCI or mild-to-moderate dementia might be suspected to be due to late-onset or autoimmune epilepsy or encephalopathy (Frisoni et al., 2024). The majority of the task force panelists did not recommend the systematic use of EEG measures in patients with MCI or mild-to-moderate dementia when clinical manifestations and structural MRI suggest a diagnosis of ADMCI or ADD.

From the perspective of the above international initiatives, rsEEG measures have limited utility as diagnostic biomarkers compared to markers considered to be disease-specific for neuropathology and neurodegeneration.

## 5. Recognizing clinical neurophysiology biomarkers of AD

This paper highlights EEG studies supporting the thesis that current clinical guidelines on AD could include measures of rsEEG rhythms reflecting cortical inhibitory/excitatory imbalance and vigilance dysfunctions in ADMCI and ADD patients. These dysfunctions significantly impact the quality of life in patients, even if these symptoms and related biomarkers do not currently allow for a differential diagnosis between AD and other neurodegenerative disorders (e.g., Lewy body diseases). For example, ADMCI and ADD patients may experience vigilance dysfunctions during activities like watching TV or hearing relaxing music, as well as alterations in the wake-sleep cycle (Jiang at al., 2024). Vigilance dysfunctions may also include mental fatigue, difficulty concentrating, and daytime drowsiness/sleepiness. This is a clinical syndrome called “mental fog,” which has recently received a lot of attention in patients suffering from long-COVID-19; these patients may share some pathological non-neurodegenerative processes (e.g., neuroinflammation) with ADMCI and ADD patients (Babiloni et al., 2024; Jiang at al., 2024). Notably, vigilance dysfunctions may be dissociated by cognitive deficits measured by neuropsychological tests. Indeed, individuals with abnormal rsEEG alpha rhythms may experience vigilance dysfunctions but perform normally on neuropsychological tests (Babiloni et al., 2024). The valuation of rsEEG rhythms in ADMCI and ADD patients with those clinical manifestations would be extremely relevant from a clinical point of view and are not well captured by standard neuropsychological tests and clinical scales for patients with neurodegenerative diseases in pathological aging.

Previous studies have found significant morning sleepiness and frequent, prolonged daytime naps in AD patients (Bonanni et al., 2005; Brzecka et al., 2018; Fang et al., 2023; Peter-Derex et al., 2015). These symptoms have been linked to cerebral beta-amyloid deposition (Lim et al., 2014), cognitive deficits (Lim et al., 2013), and functional limitations (Moran et al., 2005). Additionally, excessive daytime sleepiness has been associated with an increased risk of dementia (Leng et al., 2019), while shorter daytime naps (< 30 min) have been correlated with improved cognitive performance (Kitamura et al., 2021; Lovato and Lack, 2010; Pengsuwankasem et al., 2023). Again, the valuation of rsEEG rhythms may be relevant in ADMCI and ADD patients with those clinical manifestations.

Below, we highlight the results of selected rsEEG studies performed on ADMCI and ADD patients from independent research groups. They used various independent EEG techniques for the quantitative analysis of spatially local and global rsEEG rhythms at Berger’s frequencies from delta to alpha. Despite the heterogeneity of the methods used, all studies revealed significant abnormalities in rsEEG rhythms at one or more of those frequencies. It should be remarked that these are just a small selection of many rsEEG studies conducted in

AD patients by the following international research workgroups: the Special Interest Group on “Advanced EEG/MEG Techniques in Clinical Neurophysiology,” PDWAVES Consortium, Electrophysiology Professional Interest Area of the Alzheimer’s Association, LAC-CD, EuroLad EEG Consortium, ReDLat Consortium, eBRAIN-Health, AI-Mind, and Global Brain Consortium. It was not possible to summarize the results of all studies here; however, they have been extensively reviewed in key publications (Babiloni et al., 2020b; Colom-Cadena et al., 2020; Csernus et al., 2022; Ferreri et al., 2022; Fischer et al., 2023; Haraldsen et al., 2024; Jafari et al., 2020; Jelic and Kowalski, 2009; Kamondi et al., 2024; Koenig et al., 2020; Lam et al., 2019; Miraglia et al., 2022; Monllor et al., 2021; Moretti, 2015; Moretti et al., 2011; Rossini et al., 2020; Smailovic and Jelic, 2019; Tijms et al., 2013; Van Straaten et al., 2014).

### 5.1. The resting-state EEG (rsEEG) measures related to clinical status and neuropathological burden in AD patients as revealed by diagnostic fluid disease biomarkers

Research has consistently shown that abnormalities in rsEEG rhythms recorded in quiet wakefulness are closely associated with AD clinical status and neuropathological burden (Babiloni et al., 2021, 2020b; Rossini et al., 2020). For example, it has been shown that ADMCI and ADD patients were characterized by increased rsEEG activity in the theta range (4–7 Hz) across widespread cortical areas correlated with global cognitive performance (Musaeus et al., 2018). At the same time, reductions in alpha (8–13 Hz) and beta (14–30 Hz) rhythms were observed in posterior cortical regions compared to cognitively unimpaired individuals (Musaeus et al., 2018).

Concerning the relationships between rsEEG rhythms and AD-related neuropathology, lower CSF amyloid β42 levels – recognized as a core fluid diagnostic biomarker for AD – correlated negatively with greater rsEEG theta and delta (< 8 Hz) rhythms in individuals with ADMCI and ADD patients (Smailovic et al., 2018). Similarly, CSF phosphorylated-tau (p-tau) and total tau (t-tau) were negatively associated with global rsEEG alpha and beta rhythms in those patients (Smailovic et al., 2018). Lower CSF amyloid β42 and higher tau levels were also linked to reduced global field synchronization (GFS) computed from rsEEG alpha and beta rhythms (Smailovic et al., 2018), the GFS being a measure of the global EEG activity showing a common phase across all scalp sensors (Koenig et al., 2005). In other studies, ADMCI patients with abnormal values in the mentioned CSF diagnostic biomarkers showed higher global rsEEG delta and theta rhythms and an elevated ratio of posterior delta-theta to alpha rhythms compared to MCI patients negative for those AD biomarkers (Jovicich et al., 2019). This ratio worsened over two years, in line with global cognitive decline (Jovicich et al., 2019).

Furthermore, ADD patients presented negative associations between CSF amyloid β42 and temporal rsEEG theta rhythms and between CSF total tau and frontal-temporal rsEEG rhythms (Hata et al., 2017). High levels of CSF p-tau were related to increased temporal rsEEG theta rhythms in subjective cognitive impairment, ADMCI, and ADD patients (Musaeus et al., 2018); similar effects at rsEEG theta rhythms were reported in cognitively unimpaired older adults (Stomrud et al., 2010). These levels were also related to slowing in rsEEG alpha frequency peak in the ADMCI and ADD patients (Kramberger et al., 2013). Finally, the CSF p-tau/Aβ42 ratio was negatively associated with global rsEEG alpha rhythms (Cecchetti et al., 2021). These empirical findings support recent results of a simulation study that modeled the spread of both amyloid-β and tau proteins across a virtual human brain connectome and investigated how mathematically generated ongoing EEG dynamics were progressively affected during disease development (Alexandersen et al., 2023). By incorporating the pathological effects of both amyloid-β and tau, the model reproduced expected AD-like effects on rsEEG rhythms, including frequency slowing, early-stage hyperactivation, and late-stage hypoactivation of neuronal networks (Alexandersen et al., 2023).

Notably, we report these findings without stating that rsEEG measures may act as a surrogate for assessing brain amyloidosis and tauopathy. Instead, we stress that AD-related neuropathology may affect brain systems regulating cortical arousal and vigilance, so this is an additional reason to use those rsEEG measures in the clinical workup within the framework of precision medicine (Babiloni, 2022).

### 5.2. The rsEEG measures related to neurodegenerative burden in ADMCI and ADD patients as revealed by neuroimaging biomarkers

Previous studies have shown that abnormal rsEEG rhythms recorded in quiet wakefulness are also strongly associated with neurodegenerative burden in AD patients, as revealed by neuroimaging biomarkers (Babiloni et al., 2021, 2020b; Rossini et al., 2020). In ADMCI and ADD participants, global rsEEG delta rhythms correlated negatively with cortical gray matter volume measured by MRI, while rsEEG alpha rhythms correlated positively (Babiloni et al., 2013). In ADD patients, elevated rsEEG delta rhythms were positively associated with brain hypometabolism severity in typical AD-affected regions as measured by FDG-PET (Babiloni et al., 2016). Moreover, regional blood flow in the temporal and parietal cortical lobes, assessed by single-photon emission computerized tomography (SPECT), was negatively associated with rsEEG theta rhythms and positively associated with rsEEG alpha rhythms (Kwa et al., 1993; Müller et al., 1997; Rodriguez et al., 1999). These relationships were further influenced by subcortical white matter abnormalities revealed by MRI and chronic acetylcholinesterase inhibitor therapy (Claus et al., 2000; Rodriguez et al., 2004). Along the same line, a double-blind intravenous administration of an anti-cholinergic (scopolamine) drug over a placebo decreased rsEEG alpha rhythms and increased rsEEG delta rhythms in ADD and control participants (Neufeld et al., 1994), thus suggesting that combined measurements of rsEEG activity, neuroimaging biomarkers, and attention-motor task performances may index the integrity of cerebral cholinergic neurotransmission and predict effects of the acetylcholinesterase inhibitor therapy in AD and possibly related disorders (Neufeld et al., 1994; van Eijk et al., 2015; Schumacher et al., 2020). Notably, interhemispheric asymmetry in rsEEG and SPECT markers in ADD patients showed concordant patterns, and combining these markers improved the accuracy of the detection of AD individuals (Höller et al., 2017; Montplaisir et al., 1996). Finally, abnormalities in rsEEG delta and alpha rhythms were linked to MRI measures of subcortical vascular lesions in the white matter in ADMCI and ADD patients (Babiloni et al., 2013, 2011).

Again, we report these findings not to state that rsEEG measures may be used as a surrogate for assessing brain neurodegeneration. Rather, we want to stress that AD-related neurodegeneration may affect brain systems regulating cortical arousal and vigilance as a relevant disease dimension to assess.

### 5.3. The rsEEG measures related to brain neural dysconnectivity in ADMCI and ADD patients

As mentioned above, Edgar Adrian confirmed the existence of rsEEG alpha rhythms in humans, giving credibility to Berger’s discovery (Adrian and Matthews, 1934). However, Adrian initially disagreed with Berger on the neurophysiological interpretation of rsEEG alpha rhythms. Berger conceived rsEEG alpha rhythms as the reflection of a global, automated neurophysiological mechanism controlling mind wandering (Berger, 1938, 1929). In contrast, Adrian speculated that rsEEG rhythms were generated locally in the posterior visual cortex in relation to visual attention.

Concerning that dispute, it should be underlined that rsEEG alpha rhythms recorded at a given scalp electrode or mathematically estimated in a cortical source reflect not only an alteration of local cortical neural synchronization mechanisms but also the result of cortico-cortical functional connectivity based on signals transmitted through bundles of subcortical white matters (Babiloni et al., 2020a; Halgren et al., 2019). Along this line, both local and global neurophysiological mechanisms may be in play in the generation of rsEEG rhythms, the global mechanisms being based on cortical functional connectivity (Babiloni et al., 2020a; Halgren et al., 2019). Consequently, the rsEEG abnormalities observed in ADMCI and ADD patients may reflect AD-related cortical functional dysconnectivity. To explore this aspect, several mathematical procedures modeling cortical functional connectivity in ADMCI and ADD patients have been applied to rsEEG rhythms recorded at scalp electrodes or estimated in cortical sources (Babiloni et al., 2021, 2020b; Rossini et al., 2020). All these procedures assume that a statistical interdependence between rsEEG rhythms at scalp electrode or cortical source pairs may reflect cortical functional connectivity if the effects of head volume conduction of neural currents are adequately taken into account (Blinowska et al., 2017; Hatlestad-Hall et al., 2023; Mahjoory et al., 2017; Pascual-Marqui et al., 2014; Prado et al., 2023). In this regard, topographical estimates of cortical functional connectivity from using these techniques at scalp electrode pairs should be particularly considered with caution due to head volume conduction effects on neural currents.

Several linear and nonlinear techniques have been used to estimate cortical functional connectivity from rsEEG rhythms in ADMCI and ADD patients (Babiloni et al., 2021; Prado et al., 2023). Numerous studies in ADD patients have computed the spectral coherence of rsEEG rhythms at scalp electrode or source pairs, one of the most used linear techniques (Adler et al., 2003; Besthorn et al., 1994; Dunkin et al., 1994; Jeong, 2004; Leuchter et al., 1994, 1992; Locatelli et al., 1998). Results confirmed the abnormalities in the cortical functional connectivity modeled from rsEEG rhythms. Compared to healthy controls, ADD patients exhibited reduced frontoparietal rsEEG alpha and beta coherence, which could reflect long-range cortical functional dysconnectivity. This reduction worsened with disease progression and was more pronounced in ADD patients than in those with vascular dementia (VD). The interhemispheric decline in rsEEG spectral coherence correlated with region-specific atrophy of the corpus callosum (Pogarell, 2005). Conversely, ADD patients showed increased rsEEG spectral coherence in the delta band, which may reflect mainly deranged corticalsubcortical whitematter connectivity involving the basal ganglia and cholinergic basal forebrain (Dunkin et al., 1994; Leuchter et al., 1994, 1992). In contrast, VD patients typically presented a stable reduction in Rolandic rsEEG alpha coherence, reflecting affected short-range cortico-cortical and periventricular whitematter connectivity (Dunkin et al., 1994; Leuchter et al., 1994, 1992). These findings were further supported by studies using intrahemispheric rsEEG source functional connectivity measures, such as linear lagged coherence, which exclude zero-lag components, possibly related to head volume current conduction effects, in

ADD patients compared to those with Parkinson’s disease and Lewy body dementia (Babiloni et al., 2018). Moreover, another linear technique, the directed transfer function, modeled a reduction of cortical functional connectivity in both ADD and ADMCI patients, especially from posterior to frontal regions (Babiloni et al., 2009; Blinowska et al., 2017; Dauwels et al., 2010).

Other estimates of cortical functional connectivity from rsEEG rhythms, based on both linear and nonlinear techniques, have confirmed and expanded upon the above rsEEG spectral coherence findings in ADMCI and ADD patients. The “synchronization likelihood” is a measure of generalized (linear and nonlinear) synchronization of cortical neural activity derived from EEG signals recorded at electrode pairs (Stam et al., 2003). It quantifies the coupling between two “systems,” where the state of one system, estimated from the amplitude of EEG activity at one electrode, is mapped onto the state of the other system, estimated from the amplitude of EEG activity at the other electrode (Stam et al., 2003). In contrast, the “phase lag index” measures the asymmetry in the distribution of phase differences between two EEG signals (Stam et al., 2007). This metric may capture genuine interdependence in cortical neural activity by focusing on consistent nonzero phase lag differences, minimizing the confounding effects of volume conduction and neural current spread (Stam et al., 2007).

Methods such as “synchronization likelihood” and “phase lag index” have demonstrated reduced global interdependence of rsEEG alpha and beta rhythms in ADMCI and ADD patients relative to healthy controls, with reductions correlating with disease severity (Engels et al., 2015; Stam et al., 2003; Yu et al., 2016). Notably, frontoparietal rsEEG alpha interdependence, as measured by synchronization likelihood, has been more severely affected in ADD patients than in those with VD, consistent with findings derived from using spectral coherence techniques (Babiloni et al., 2004). Finally, other nonlinear EEG techniques have successfully been used to measure decreased complexity, increased entropy, and reduced information transmission among cortical areas in ADD and ADMCI patients, providing further support to the thesis of abnormal dynamics of cortical neural synchronization and functional connectivity in the continuum of AD course (Dauwels et al., 2010; Jeong, 2004; Simmatis et al., 2024; Sun et al., 2020).

A promising avenue of research uses the above EEG estimates of the cortical functional connectivity as input to graph theory procedures modeling the topology (i.e., network structure with nodes and edges as node connectors) of the AD-related derangement of that connectivity (Reijneveld et al., 2007; Tijms et al., 2013). Several rsEEG studies showed that healthy persons are characterized by a resilient network structure called “small world,” defined as a balanced pattern of short-trait graph connectors forming local networks (i.e., clusters) and long-trait graph connectors (i.e., hubs) forming global networks (De Haan et al., 2009; Franciotti et al., 2019; Frantzidis et al., 2014; Stam et al., 2007; Supekar et al., 2008; Vecchio et al., 2024, 2016, 2014a,b). In general, the results of this approach suggest that the “small-world” network may reflect efficient interdependence between local and global control mechanisms underpinning the cortical inhibitory/excitatory balance generating rsEEG rhythms in healthy adults (Reijneveld et al., 2007; Stam et al., 2007; Tijms et al., 2013).

Regarding clinical applications of the above concepts, previous rsEEG studies have basically demonstrated reduced long-trait graph connectors in ADMCI patients, with even more pronounced reductions in ADD patients (De Haan et al., 2009; Franciotti et al., 2019; Frantzidis et al., 2014; Stam et al., 2007; Supekar et al., 2008; Vecchio et al., 2024, 2016, 2014a,b). This has been interpreted as a shift from the resilient “small world” network structure toward a more random topology of functional connectivity, supporting the hypothesis that AD progression significantly disrupts brain neural networks (De Haan et al., 2009; Franciotti et al., 2019; Frantzidis et al., 2014; Stam et al., 2007; Supekar et al., 2008; Teipel et al., 2016; Vecchio et al., 2024, 2016, 2014a,b). This functional change may be associated with the deterioration of subcortical white matter integrity. For instance, studies have shown that reduced callosal connections between the cerebral hemispheres, as measured by MRI tractography, correlated with reduced long-trait connectors in ADMCI and ADD patients compared to healthy controls (Vecchio et al., 2015).

Beyond “small world” properties, additional graph metrics, such as network modularity and connector directionality, have been explored to model the hierarchical and complex organization of local and global cortical networks in AD and ADMCI patients (Abazid et al., 2021; Franciotti et al., 2021, 2019; Lopez et al., 2023; Peraza et al., 2018). Along this line, an interesting study in MCI patients revealed the relationship between rsEEG rhythms, vigilance function, and topological markers of network centralization of the information processing from the phase lag index of rsEEG rhythms at electrode pairs as estimates of cortical functional connectivity (Kavcic et al., 2021; Požar et al., 2023). The vigilance level was modulated by a visual motion direction discrimination task performed between two rsEEG recordings (Kavcic et al., 2021; Požar et al., 2023). MCI patients showed a greater amplitude reduction of posterior rsEEG alpha rhythms after the task compared to cognitively unimpaired older persons (Kavcic et al., 2021). They also showed an increased betweenness centrality of the graph networks, maybe as a mechanism compensating for the loss of rsEEG rhythms (Požar et al., 2023).

Overall, these graph theory metrics have provided significant insights into how AD disrupts the topological structure of cortical functional connectivity. However, an important need in the field is an international consensus initiative to standardize procedures to produce and report those metrics (Miljevic et al., 2022). This includes defining the optimal preprocessing pipeline, number of scalp electrodes, EEG band limits, interdependence measures for rsEEG rhythms at scalp electrodes and source pairs, and statistical thresholds for graph theory indexes (Allouch et al., 2023; Babiloni et al., 2020a; Kabbara et al., 2023; Lopez et al., 2023; Miljevic et al., 2022). Such standardization will be valuable to reduce variability across studies that have reported topological effects from estimates of AD-related cortical functional dysconnectivity in different EEG frequency bands. Susceptibility to volume conduction can be reduced by estimating cortical functional connectivity from scalp-recorded EEG signals. One approach involves using spatial filters that attenuate low spatial frequencies in EEG voltage distributions, such as surface Laplacian estimates, without requiring an explicit mathematical model of EEG sources (Srinivasan et al., 2007). This method effectively minimizes the impact of volume conduction on spectral coherence estimates of EEG activity recorded at scalp electrode pairs (Srinivasan et al., 2007). Another approach estimates cortical functional connectivity at the source level, relying on mathematical and biophysical models of both head volume conductor and cortical sources from scalp-recorded EEG activity to solve the so-called “EEG inverse problem” (Biscay et al., 2018; Pascual-Marqui et al., 2014). Since the inverse problem lacks a unique solution, this estimation should ideally incorporate structural brain connectivity priors (Hammond et al., 2013) and employ multiple estimators or assumptions to compare solutions derived from different modeling approaches. (Hatlestad-Hall et al., 2023; Mahjoory et al., 2017). One of the methods to reduce the common source effect in estimating cortical functional connectivity from rsEEG activity—which can lead to spurious connections in bivariate measures— is the use of a multivariate autoregressive estimator such as the directed transfer function (Babiloni et al., 2009; Biscay et al., 2018; Blinowska et al., 2017, 2013; Dauwels et al., 2010; Pascual-Marqui et al., 2014).

The results of this section agree with Berger’s view that rsEEG alpha rhythms reflect global neurophysiological mechanisms controlling vigilance in quiet wakefulness.

### 5.4. The rsEEG biomarkers for classification and clinical predictions of ADMCI and ADD patients

Berger discovered and described prominent rsEEG alpha rhythms and their variations in response to experimental conditions or neurological and psychiatric diseases through visual analysis at the individual level (Berger, 1929). His publications featured rsEEG rhythms of individual cases, illustrating the relationship between changes in these rhythms and vigilance/consciousness levels during recording (Berger, 1938, 1929).

Following Berger’s perspective, numerous studies have applied various quantitative rsEEG measures derived from the mentioned linear and nonlinear techniques as input features to machine learning (ML) models for analysis at the individual level in ADMCI and ADD patients. These models aim to classify ADMCI or ADD patients vs. healthy control adults and predict clinical outcomes in those patients at follow-ups. The underlying assumption is that disease effects on rsEEG rhythms may be detected at the individual level. This is important for developing reproducible biomarkers for clinical workup, capable of characterizing ADMCI and ADD individuals through their rsEEG rhythms and providing stratification or prognostic information within a mode of precision medicine.

Table 1 summarizes key data from 36 example studies focused on these objectives. While a detailed analysis of their findings and methodologies is beyond the scope of this paper, the core messages are as follows: the average accuracy of detecting ADMCI and ADD patients compared to healthy controls based on rsEEG measures and ML tools was > 85 %, and the average accuracy of predicting clinical AD status at follow-ups was about 80 %.

These findings are not reported to propose rsEEG biomarkers for a diagnosis of AD in the clinical workup, as they are not direct measures of abnormal brain amyloidosis-tauopathy and “accurate classification” does not mean “diagnosis”. Rather, they are reported to underline that rsEEG measures, when used as input features into ML tools, can provide insights into neurophysiological abnormalities in ADMCI and ADD patients at the individual level. When cross-validated, rsEEG measures have the potential to aid clinical decision-making by accurately classifying ADMCI and ADD patients based on the degree of abnormalities in their rsEEG rhythms and the associated dysregulation of cortical arousal and vigilance. This information may guide the intervention to normalize that dysregulation in ADMCI and ADD patients.

Aside from the above considerations, ML algorithms offer an additional opportunity. They may allow the integration of rsEEG with neuroimaging and fluid biomarkers of AD for the construction of a virtual brain model in AD (Schirner et al., 2023, 2022). For clinical adoption, transparent and explainable models are valuable, as healthcare professionals need to understand how specific biomarkers contribute to diagnostic decisions.

### 5.5. The EEG activity revealing epileptiform activity in ADMCI and ADD patients

Berger showed the strict relationship between rsEEG rhythms and vigilance level in patients with epilepsy, comparing those rhythms before and immediately after a seizure with loss of consciousness (Berger, 1938). In the last decades, several studies have shown an increased risk of overt epileptic seizures in ADMCI and ADD patients, with faster clinical deterioration compared to those patients without epilepsy (Horváth et al., 2017, 2016; Kamondi et al., 2024; Vossel et al., 2016). Furthermore, convulsive seizures are ten times more frequent in ADD patients than in the general population (Horváth et al., 2016), and a clinical diagnosis of epilepsy is 87 times more common in patients with early-onset ADD than those with late-onset disease (Scarmeas et al., 2009).

Additionally, cognitive impairment appears 5.5 years earlier in ADD patients with epileptiform activity compared to those without that activity (Vossel et al., 2013). Most epileptiform activity is identified during extended EEG recordings, especially during non-rapid eye movement (NREM) sleep, suggesting the utility of long rsEEG recording sessions for detecting the presence of epileptiform activity in ADMCI and ADD patients (Horváth et al., 2017).

ADMCI and ADD patients may also present abnormalities in cortical neural synchronization mechanisms and inhibitory/excitatory balance associated with ***subclinical*** epileptiform activity (SEA) as spikes and sharp waves (Brunetti et al., 2020; Horvath et al., 2021; Musaeus et al., 2023; Vossel et al., 2016). SEA is a kind of electrophysiological manifestation detected in people who have never had clinically diagnosed epileptic seizures (Chatrian et al., 1974; Kane et al., 2017; Noachtar et al., 1999). The nature and frequency of SEA in ADMCI and ADD patients are debated (Brunetti et al., 2020; Vossel et al., 2016). However, it has been shown that SEA is associated with faster cognitive decline in ADD patients compared to those who do not show it (Horvath et al., 2021; Vossel et al., 2016).

Notably, ADMCI and ADD patients showing SEA are characterized by abnormal brain electromagnetic rhythms even in recording periods without epileptiform activity. Indeed, reduced alpha rhythms and enhanced delta rhythms have been reported in resting-state MEG (rsMEG) recordings from ADD patients (Ranasinghe et al., 2022). In addition, increased posterior rsEEG delta rhythms have been observed in ADMCI patients with SEA compared to those without it (Babiloni, 2022). This epileptogenic activity may affect brain neural networks that generate not only low-frequency but also high-frequency brain neural rhythms. Indeed, rsMEG studies showed abnormalities even in gamma rhythms (around 40 Hz) in ADMCI and ADD patients with subclinical epileptiform activity (Cuesta et al., 2022; Prabhu et al., 2024).

A new promising aspect of cortical inhibitory/excitatory imbalance in ADMCI and ADD patients is the analysis of the “aperiodic” component of the rsEEG power density spectrum, defined by the slope of the reduction in the EEG power density from lower to higher frequencies. It has been shown that GABAergic agonists administered to healthy volunteers induce loss of vigilance and anesthesia in association with the disappearance of the “periodic” EEG alpha power peak and a significant reduction in the slope of that “aperiodic” component (Brake et al., 2024). This analysis has recently been applied in AD patients and controls with mixed results (Azami et al., 2023; Burelo et al., 2024; Kopčanová et al., 2024; Wang et al., 2023) but may be discriminating in those with epileptiform activity.

The above findings suggest that quantitative neurophysiological measures from rsEEG and rsMEG may be sensitive to brain network hyperexcitability in ADMCI and ADD patients, including the cases showing SEA. Incorporating these measures into the clinical workup could help to identify patients who may benefit from anti-seizure medication to treat cortical hyperexcitability and mitigate vigilance dysfunctions and, possibly, cognitive deficits (Kamondi et al., 2024). Furthermore, EEG measures may guide AD patients’ stratification in clinical studies to test the efficacy of (non) pharmacological interventions for mitigating cortical hyperexcitability and vigilance dysfunctions (Kamondi et al., 2024).

The utility of EEG recordings to detect clinically relevant epileptiform activity in MCI or mild-to-moderate dementia patients has recently been acknowledged by a Delphi consensus initiative from several European neuroscience societies (Frisoni et al., 2024). However, these EEG recordings have been recommended only for patients suspected of having undiagnosed late-onset or autoimmune epilepsy but not for all patients with suspected ADMCI or ADD status (Frisoni et al., 2024). This expert panel suggests that the presence of clinical and subclinical epileptiform activity should be evaluated even in diagnosed ADD and ADMCI patients when clinicians suspect the presence of such activity.

### 5.6. An EEG pathophysiological marker in the assessment of ADMCI and ADD patients

**Fig. 2.**
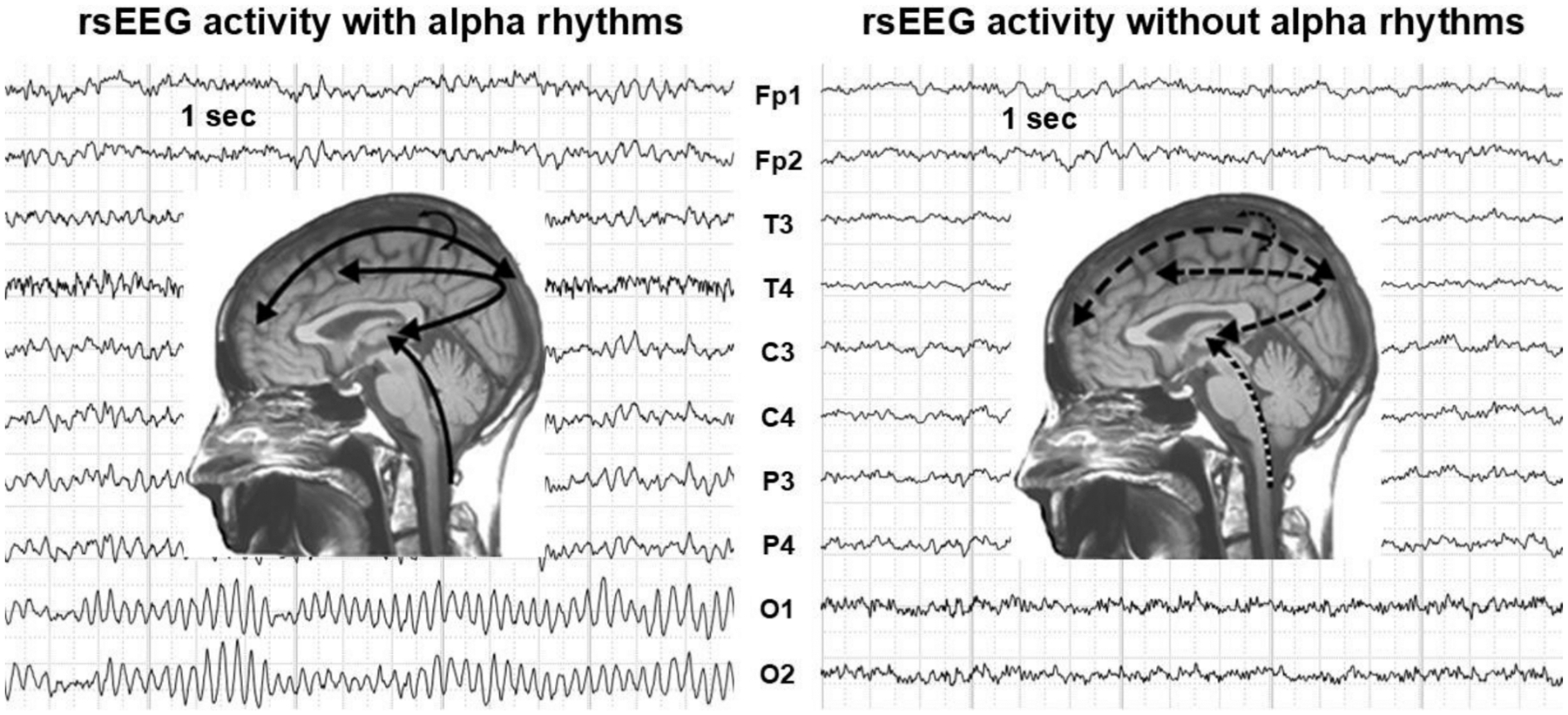
A proposed neurophysiological model for the generation of rsEEG alpha rhythms in cognitively unimpaired older adults (Left in Figure) and Alzheimer’s disease (AD) patients (Right in Figure). In the normal brain of cognitively unimpaired older adults in quiet wakefulness, dominant EEG rhythms are observed in the alpha frequency range (8–12 Hz), reflecting the spontaneous synchronization around 10 Hz of neural networks involved in regulating global arousal and levels of vigilance/consciousness. These networks encompass neural populations in the cerebral cortex, thalamus, basal forebrain, and brainstem, including glutamatergic, cholinergic, dopaminergic, and other components of the ascending reticular activating system. The normal brain generates rsEEG delta (< 4 Hz) and theta (4–7 Hz) rhythms with low amplitude. In the AD brain, there is a reduction in the amplitude of rsEEG alpha rhythms (i.e., tonic background desynchronization) accompanied by an abnormal increase in the amplitude of rsEEG delta (< 4 Hz) and theta (4–7 Hz) rhythms. The shift of these neurophysiological oscillatory mechanisms toward slower rsEEG rhythms is thought to reflect a state of thalamocortical disconnection with disruptive effects on background cortical functional connectivity in quiet wakefulness and impacting vigilance regulation. The rsEEG traces represented in this Figure are available as uncredited, public domain, thanks to Wikimedia Commons. File for the rsEEG activity with alpha rhythms (Left in Figure): “Human_EEG_with_prominent_alpha-rhythm.png”- https://commons.wikimedia.org/wiki/File:Human_EEG_with_prominent_alpha-rhythm.png. File for the rsEEG activity without alpha rhythms (Right in Figure): “Human_EEG_without_prominent_alpha-rhythm.png” – https://commons.wikimedia.org/wiki/File:Human_EEG_without_prominent_alpha-rhythm.png.

The abnormalities in rsEEG rhythms reported above in ADMCI and ADD patients may stem from deranged oscillatory activity in neural populations within thalamocortical and corticothalamic circuits (De Munck et al., 2007; Halgren et al., 2019; Knaut et al., 2019). These circuits are modulated by the brainstem ascending reticular activating system and projections from the basal forebrain, which utilize neurotransmitters such as glutamate, acetylcholine, dopamine, and others (Babiloni et al., 2020a, 2020b; Crunelli et al., 2015; Dey et al., 2016; Hughes and Crunelli, 2005; Moruzzi and Magoun, 1949). In this neurophysiological model (Fig. 2), the shift from alpha to delta/theta frequencies in the rsEEG activity recorded from ADMCI and ADD patients may represent a state of thalamocortical disconnection from these ascending systems, leading to alterations in background cortical functional connectivity and vigilance regulation. For example, rsEEG alpha rhythms have been shown to be reduced in ADMCI patients in relation to lesions in the cholinergic tracts from the basal forebrain to the cerebral cortex (Babiloni et al., 2009). Furthermore, the reactivity of rsEEG alpha rhythms from eyes closed to open was reduced in ADD patients compared to controls in association with reduced volume of cholinergic basal forebrain neurons (Schumacher et al., 2020).

As AD progresses, the systems responsible for generating rsEEG rhythms may be directly impacted by ADrelated neuropathology and neurodegeneration at both cortical and subcortical levels, including the brain’s ascending reticular formation and hypothalamic systems involved in sleep-wake cycle regulation (Ehrenberg et al., 2023). In addition, these systems may also be indirectly affected by concurrent processes such as neuroinflammation, immune reactivity, and cerebrovascular lesions (Jack et al., 2024; Jiang at al., 2024).

As mentioned above, we posit that altered rsEEG rhythms may serve as neurophysiological biomarkers of a class of common non-cognitive symptoms in ADMCI and ADD patients, such as mental fatigue, difficulties in maintaining concentration over several minutes, excessive daytime sleepiness, and others that are typically summarized in the general concept of “mental fog” in the recent literature on long-COVID-19 patients (Jiang at al., 2024). Such symptoms can be caused by AD-triggered (e.g., neurodegeneration and neuroinflammation) or concomitant (e.g., cerebrovascular lesions) causes (Jiang at al., 2024) and are clinically relevant as they may predict patient’s difficoulties in everyday activities like watching relaxing TV programs, hearning resting music, etc. Given the significant impact of these non-cognitive symptoms on the quality of life of AD patients and their parents, we propose that current clinical guidelines be expanded to include pathophysiological “P” biomarkers derived from rsEEG activity in the clinical workup of patients with MCI or mild-to-moderate dementia due to AD (Table 2). “P” biomarkers could offer valuable insights into the dysfunction of neuromodulatory subcortical-cortical systems that regulate cortical inhibitory/excitatory balance and vigilance, both of which are valuable for daily functioning in AD patients. Incorporating these markers into the clinical workup could improve the monitoring of AD progression in relation to non-cognitive symptoms and provide new endpoints for interventions with targeted therapies within the framework of precision medicine (Hampel et al., 2023, 2019, 2018).

## 6. Recommendations to promote the use of rsEEG for the assessment of ADMCI and ADD patients

The expert panel of this initiative reached a consensus that rsEEG biomarkers of cortical inhibitory/excitatory imbalance and vigilance/consciousness level dysfunctions should be used in the clinical workup of ADMCI and ADD patients. Notably, the state-of-the-art shows several candidate rsEEG measures from linear and nonlinear techniques for AD patient management or clinical trials without a clear indication of the most valid and reliable. Therefore, international sponsors of research should invest in international multicenter studies to compare the output of the methods that showed the best results in previous rsEEG studies performed in ADMCI and ADD patients. In this regard, the expert panel agreed on the following recommendations on good experimental practices for future studies. These recommendations are based on previous guidelines and recommendations from the International Federation of Clinical Neurophysiology (Babiloni et al., 2020a; Cole and Kamondi, 2023; De Weerd and Clarenbach, 1999; Nuwer et al., 1999; Seeck et al., 2017). They are also based on recent qualified international initiatives aimed at defining criteria to harmonize and optimize procedures for collecting and analyzing rsEEG data for clinical applications (Bigdely-Shamlo et al., 2020; Jaramillo-Jimenez et al., 2024; Li et al., 2022).

### 6.1. Before and during the rsEEG recording

Future multicenter rsEEG studies should be prospective and longitudinal and begin with a rigorous standardization phase to ensure consistency in experimental design and procedures. This includes (1) the collection of information about their participant’s general sleep quality and sleepiness during the daytime, use of chronic medication and psychoactive substances, and the sleep quality and psychoactive substances taken the day before the EEG experiment; (2) annotation of experimental equipment and experimental procedures and settings, including instructions for participants regarding psychophysiological mode during EEG recording (e.g., mind wandering); and (3) the control of the recording environment for noise and light and annotations of relevant information on the development of the EEG experiment.

EEG recordings should be planned in the late morning and ideally employ more than 32 scalp electrodes (1020, 10–10, or 10–5 systems) with control sensors (e.g., electrocardiographic and electrooculographic electrodes). This setup is appropriate for clinical workup and exploratory studies of rsEEG sources in ADMCI and non-ADMCI patients using low-resolution source estimation techniques. For detailed brain source connectivity analysis from rsEEG rhythms, more than 48 electrodes are recommended (Hatlestad-Hall et al., 2023). For clinical workup and quantitative analysis of rsEEG rhythms at the scalp electrode, 25 sensors according to the updated 10–20 system may apply (Seeck et al., 2017).

The duration of the resting-state condition with eyes closed and open should be standard (3–5 min each). When possible or for specific clinical indications, this condition should be followed by hyperventilation and photic stimulations to test susceptibility to epileptiform activity, even in ADMCI and ADD patients without any previous report of epilepsy. Neurophysiological mechanisms underpinning cortical inhibitory/excitatory imbalance and abnormal transitions from quiet vigilance to light sleep may also be explored with an additional condition of EEG recording for > 30 min with eyes closed and the instructions allowing participants to fall asleep (Ulke et al., 2019). This period may be prolonged to several hours and include night sleep recordings in ADMCI and ADD patients with suspected epileptiform activity or even late-onset epilepsy (Horváth et al., 2017; Lam et al., 2019; Vossel et al., 2016, 2013).

### 6.2. After the rsEEG recording

The data format (e.g., BIDS) and preprocessing (e.g., re-referencing, artifactual channel interpolation) should be standardized (Babiloni et al., 2021; Halford et al., 2023; Pernet et al., 2019).

Clinical experts should visually review rsEEG data blind to the participant’s diagnosis both before and after preprocessing for artifact detection and removal, as unsupervised, automatic data quality assessment procedures for large rsEEG databases are promising but have been not yet validated by international consensus initiatives (Zhao et al., 2023).

When studying rsEEG alpha rhythms in ADMCI and ADD patients, researchers should account for the effects of the reference electrode (Dong et al., 2021), individual alpha frequency peaks (IAFp), and alpha sub-bands (low and high frequencies), as these patients may exhibit varying degrees of alpha rhythm slowing (Klimesch, 1999). An automated approach for defining alpha frequency bands, such as frequency principal components analysis (Barry et al., 2020), could offer more nuanced insights given the complexity of alpha oscillations.

Cortical source activity and connectivity estimates of rsEEG rhythms should ideally be validated by at least two independent mathematical methods, as no unique EEG/MEG inverse source solution exists (HatlestadHall et al., 2023; Mahjoory et al., 2017).

EEG measures should be integrated into comprehensive brain aging and AD models that incorporate multimodal, genetic, biophysical, and neuropathological measures to explore the multi-scale processes underlying cortical excitation/inhibition imbalance and vigilance dysfunctions (Alexandersen et al., 2023; Ibanez et al., 2024; Schirner et al., 2023, 2022; Smits et al., 2016). This could include co-registrations with structural and functional MRI data, along with advanced Bayesian and machine-learning models, in accordance with the NIA-AA (Jack et al., 2024, 2018) and the European Neuroscience intersocietal guidelines (Frisoni et al., 2024). Furthermore, another promising approach is based on investigating EEG responses evoked by non-invasive brain stimulations, allowing effective measures of cortical overexcitability in ADD patients (Casula et al., 2023; Maiella et al., 2024).

Statistical models should also consider key AD risk factors such as diet, physical activity, cardiometabolic health, ethnoracial and socio-economic factors, and national and economic structural inequality to provide a more comprehensive understanding of brain health, including those from underrepresented populations. Recent EEG studies on pathological aging led by the LAC-CD Consortium exemplify this approach (Baez et al., 2023; Hernandez et al., 2024; Moguilner et al., 2024).

Finally, international collaboration, software development, anonymized data sharing, and open science are crucial for advancing the standardization and validation of rsEEG biomarkers in ADMCI and ADD patients.

## 7. Overview and conclusions

In this “centenary” paper, an expert panel revisited Hans Berger’s pioneering discovery of human rsEEG rhythms in 1924 and their association with vigilance and consciousness levels. Berger envisioned EEG as a critical tool for understanding brain function and dysfunction, including what was previously named “senile dementia.” Along this line, the expert panel reached a consensus on the thesis that Berger’s rsEEG rhythms may reflect cortical inhibitory/excitatory imbalance and vigilance dysfunctions in ADMCI and ADD patients—clinical manifestations that significantly may impact quality of life. The alteration of these rhythms could serve as a surrogate “symptomatic” biomarker for such clinical manifestations, even if they are not disease-specific and cannot support a differential diagnosis of AD. These biomarkers could be used in both clinical workup (individual level) and drug discovery pathways (group level), especially in ADMCI and ADD patients showing clinically relevant vigilance dysfunctions.

The expert panel examined the status of rsEEG measures in light of recent international clinical guidelines— published 100 years after Berger’s discovery (Frisoni et al., 2024; Jack et al., 2024). These guidelines outline the use of fluid and neuroimaging biomarkers for assessing patients across the clinical continuum from MCI to mild-to-moderate dementia (Frisoni et al., 2024; Jack et al., 2024, 2018). Despite extensive research reporting significant abnormalities in rsEEG rhythms, these guidelines currently assign only marginal value to rsEEG biomarkers.

A narrative review of the findings of selected studies showed converging evidence of rsEEG abnormalities in ADMCI patients and ADD patients, particularly at delta, theta, and alpha frequencies. These abnormalities have been demonstrated consistently across multiple and independent multicenter studies using various analytic techniques at both group and individual levels. Moreover, these abnormalities correlate with established AD biomarkers of neuropathology and neurodegeneration and align with models of cortical overexcitability and hyper-synchronization, as reflected by subclinical epileptiform activity in a significant number of cases.

The expert panel advocates for integrating rsEEG measures as “pathophysiological biomarkers” into the clinical assessment of ADMCI and ADD patients, especially those with substantial vigilance dysfunctions, highlighting their potential to capture significant neurophysiological changes related to cortical inhibitory/excitatory imbalance at the individual level. Additionally, international efforts should focus on comparing the outcome of the most promising EEG techniques to qualify optimal rsEEG biomarkers for drug discovery pathways, particularly for the treatment of cortical inhibitory/excitatory imbalance and vigilance dysfunctions in ADMCI and ADD patients.

Berger’s vision of EEG’s role in dementia due to pathological brain aging is still actual in the framework of precision medicine.

## Figures and Tables

**Table 1:** Machine-learning classification/prediction studies using resting-state electroencephalographic (EEG) markers in Alzheimer’s disease (AD) patients.

Machine-learning classification/prediction studies using rsEEG markers in AD				
Paper	Application	Tool	Participant	Accuracy
Prichep et al., 2005	Prediction at 7–9 year follow-up	Logistic regression	37 Healthy: 17 Healthy stable, 20 Healthy with a decline at follow-up	90 %
Missonnier et al., 2006	Prediction at 1 – year follow-up	Regression analysis	24 MCI: 11 MCI stable and13 MCI with a decline at follow-up	%
Buscema et. al., 2010	Prediction at 1 – year follow-up	ANN	143 MCI: 92 MCI-stable and 51 MCI converted toADD at follow-up	85 %
Poil et al., 2013	Prediction at 2 – years follow-up	Logistic regression,	330 MCI: 322 MCI stable and 8 MCI converted toADD at follow-up	85 %
Mazaheri et al., 2017	Prediction within 3 – year follow-up	Regression analysis	20 MCI: 10 MCI stable and10 MCI converted to ADD at follow-up	%
Vecchio et al., 2018	Prediction at 1 – year follow-up	Polynomial regression	145 MCI: 71 MCI stable and 74 MCI converted toADD at follow-up	61 %
Tait et al., 2020	Prediction at 4 – year follow-up		11 MCI: 7 MCI stable and4 MCI converted to ADD at follow-up	%
Chu et al., 2023	Prediction within 3 – year follow-up	LogitBoost, Bagging,Gentle adaptive boosting, Decision tree, SVM, Naïve Bayes, and KNN	72 MCI: 36 MCI stable and36 MCI converted to ADD at follow-up	%
Abasolo et al., 2008	Diagnostic classification	LDA	11 Health vs. 11 ADD.	95 %
Trambaiolli et al.,2011	Diagnostic classification	SVM	19 Healthy vs. 16 ADD	87 %
Aghajani et al., 2013	Diagnostic classification	Linear SVM, LOOCV	17 Healthy vs. 17 ADD	84 %
McBride et al., 2015	Diagnostic classification	SVM	15 Healthy vs. 16 MCI	91 %
Simons et al., 2015.	Diagnostic classification	LDA	11 Healthy vs. 11 ADD	77 %
Morabito et al., 2016	Diagnostic classification	SVM, MLP-NN	23 Healthy, 23 MCI vs. 23ADD	85 % (Healthy vs ADD) 85 % (Healthy vs MCI) 78 % (MCI vs ADD)
Blinowska et al., 2017	Diagnostic classification	Directed TransferFunction, Mahalanobis Distance	42 Healthy vs. 42 AD	86 %
Trambaiolli et al.,2017	Diagnostic classification	SVM	12 Healthy vs. 22 ADD	91 %
Triggiani et al., 2017	Diagnostic classification	ANN	100 Healthy and 120 CE	77 %
Ruiz-Gómez et al., 2018	Diagnostic classification	LDA, QDA, MLP	37 Healthy vs. 37 ADD	82 %
Farina et al., 2020.	Diagnostic classification	Penalized logistic regression	198 Healthy, 134 MCI vs. 118 ADD	76 % (Healthy vs ADD) 67 % (MCI vs ADD)
Vecchio et al., 2020	Diagnostic classification	SVM	120 Healthy vs. 175 ADD	95 %
Ieracitano et al., 2020	Diagnostic classification	AE, MLP, SVM, and LR	63 Healthy, 63 MCI vs. 63ADD	92 % (Healthy vs ADD) 91 % (Healthy vs MCI) 84 % (MCI vs ADD)
Nobukawa et al., 2020	Diagnostic classification	SVM	18 Healthy vs. 16 ADD	74 %
Safi and Safi, 2021	Diagnostic classification	SVM, KNN and RLDA	35 Healthy vs. 31 mild AD, and 20 moderate AD	%
Li et al., 2021	Diagnostic classification	SVM	21 Healthy vs. 28 MCI	86 %
Miltiadous et al., 2021	Diagnostic classification	DT, RF, ANN, SVM, Naïve Bayes, and kNNs	8 Healthy vs. 10 ADD	78 %
Alessandrini et al., 2022	Diagnostic classification	PCA, RNN	15 Healthy vs. 20 ADD	97 %
García-Pretelt et al., 2022	Diagnostic classification	SVM	33 Healthy v. 27 Asymptomatic FamilialAD Carriers	83 %
Ding et al., 2022	Diagnostic classification	P-En, S-En, W-En, and LZ	113 Healthy, 116 MCI vs.72 ADD	80 % (Healthy vs ADD) 71 % (Healthy vs MCI) 64 % (MCI vs ADD)
Jiang et al., 2022	Diagnostic classification	SVM	152 Healthy vs. 184 MCI	84 %
Perez-Valero et al., 2022	Diagnostic classification	Scikit-learn Python, SVM, logistic regression	7 Healthy vs. 7 ADD	86 %
Chu et al., 2023	Diagnostic classification	LogitBoost, Bagging,Gentle adaptive boosting, Decision tree, SVM, Naïve Bayes, and KNN	51 Healthy, 42 MCI vs. 61 ADD	81 % (Healthy vs MCI) 86 % (MCI vs ADD)
Kim et al., 2023a	Diagnostic classification	SVM, Logistic, KNN, NB,RF, AdaBoost, GBM and XGBoost	20 MCI Aβ positive vs. 19 MCI Aβ negative	84 %
Kim et al., 2023b	Diagnostic classification	Ensemble model(KNN, RF, SVM, ANN)	459 Healthy vs. 417 MCI,311 ADD & VD	74.6 % (Average) 89 % (Healthy) 75 % (MCI) 85 % (ADD & VD)
Parreño Torres et al., 2023	Diagnostic classification	SVM, BLD, DT, GNB, KNN, and RT.	261 Healthy vs. 201 ADD	93 %
Said and Göker, 2023	Diagnostic classification	Bi-LSTM DT, SVM, KNN	16 Healthy vs. 18 MCI	98 %
Simfukwe et al., 2023	Diagnostic classification	Regression analysis	269 Healthy, 356 MCI vs. 265 ADD	83 % (Healthy vs ADD) 81 % (Healthy vs MCI)

AE: Autoencoder.

ANN: Artificial Neural Networks.

Bi-LSTM: Bidirectional Long Short-Term Memory.

BLD: Bayesian Linear Discriminant.

CNN: Convolutional Neural Network.

DT: Decision Tree.

ET model: Eye-tracking Model.

GBM: Gradient Boosting Machine.

KNN: K-Nearest Neighbor Algorithm LDA: Linear Discriminant Analysis.

LOOCV: Leave-One-Out Cross-Validation

LR: Logistic Regression

LZ: Lempel-Ziv.

MLP-NN: Multi-Layer Perceptron neural networks.

NB: Naive Bayes.

PCA: Principal Component Analysis.

P-En: Permutation Entropy.

PSEN1-E280A: Missense Mutation in Presenilin 1 associated with early-onset Familial ADD QDA: Quadratic Discriminant Analysis.

RF: Random Forest.

RLDA: regularized linear discriminant analysis.

RNN: Recurrent Neural Network.

S-En: Sample Entropy.

SVM: Support Vector Machine VD: Vascular Dementia.

W-En: Wavelet Entropy.

**Table 2:** Theoretical proposal for an Alzheimer’s disease (AD) model (ATPINO) and the biomarkers for in vivo measurements of the model dimensions. The model dimensions include amyloidosis (A), tauopathy (T), pathophysiology (P), Inflammation (I), and neurodegeneration (N). The disease processes within those dimensions produce a clinical output (O) involving vigilance, sleep-wake cycle, cognitive functions, and abilities in the activities of daily living. Legend: CSF, cerebrospinal fluid; PET, positron emission tomography; EEG, electroencephalography; ERO, event-related EEG oscillations; ERP, event-related potentials; MRI, magnetic resonance imaging; FDG-PET, fluorodeoxyglucose-positron emission tomography. CSF and PET biomarkers are reported with their molecular codes.

Alzheimer’s disease model and biomarkers			
**A**	Amyloid		CSF Aβ42 or Aβ42/Aβ40 ratio PET [11C]-PiB, [18F]-Florbetapir
**T**	Taupathy		CSF phosphorylated tau Tau PET
**I**	Inflammation		CSF sTREM2, IL-6, IL-1β, TNF-α PET [18F]-DPA-714, [11C]-PBR28
**P**	Pathophysiology		Resting-state eyes-closed/open EEG ERO/ERP
**N**	Neurodegeneration		Structural MRI FDG-PET
**O**	Output	Vigilance and Sleep/Wake	Neuropsychological tests Psychophysics
		Cognition	Clinical scale
